# Dehydroeffusol inhibits hypoxia-induced epithelial–mesenchymal transition in non-small cell lung cancer cells through the inactivation of Wnt/β-catenin pathway

**DOI:** 10.1042/BSR20194284

**Published:** 2020-05-28

**Authors:** Haitao Wei, Feng Zhang, Jiali Wang, Min Zhao, Tao Hou, Li Li

**Affiliations:** 1 Department of Thoracic Surgery, Huaihe Hospital of Henan University, Kaifeng 475000, P.R. China; 2 Operating Room, Huaihe Hospital of Henan University, Kaifeng 475000, P.R. China; 3 Department of Respiratory, Huaihe Hospital of Henan University, Kaifeng 475000, P.R. China; 4 College of Nursing and Health, Henan University, Kaifeng 475001, P.R. China

**Keywords:** Dehydroeffusol (DHE), epithelial-mesenchymal transition (EMT), hypoxia, non-small cell lung cancer (NSCLC), Wnt/β-catenin pathway

## Abstract

Dehydroeffusol (DHE) is a phenanthrene compound that possesses anti-tumor activity. However, the effect of DHE on non-small cell lung cancer (NSCLC) has not been investigated previously. Therefore, the objective of our study was to explore the role of DHE in NSCLC and the underlying mechanism. Our results showed that DHE significantly inhibited the cell viability of A549 cells in a dose- and time-dependent manner under normoxic condition. Moreover, A549 cells were more sensitive to DHE under hypoxic condition compared with the A549 cells cultured in normoxic condition. Hypoxia-induced increased migration and invasion abilities were mitigated by DHE in A549 cells. Treatment of DHE caused increased E-cadherin expression and decreased N-cadherin expression in hypoxia-induced A549 cells. DHE also suppressed hypoxia-induced increase in both protein and mRNA levels of hypoxia inducible factor-1α (HIF-1α) expression in A549 cells. Furthermore, DHE inhibited hypoxia-induced activation of Wnt/β-catenin pathway in A549 cells. The inhibitory effect of DHE on hypoxia-induced EMT was reversed by LiCl, which is an activator of Wnt/β-catenin signaling pathway. In conclusion, these findings demonstrated that DHE prevented hypoxia-induced EMT in NSCLC cells by inhibiting the activation of Wnt/β-catenin pathway, suggesting that DHE might serve as a therapeutic target for the NSCLC metastasis.

## Introduction

Lung cancer is one of the most deadly cancers in the world [1]. Lung cancer is majorly bifurcated into non-small cell lung cancer (NSCLC) and small cell lung cancer (SCLC). NSCLC is the main type of lung cancer represented by 85% of the cases [2]. Advances in clinical research have improved the treatment options available for NSCLC; however, more than 50% of the NSCLC patients die within one year of diagnosis and the 5-year survival rate is less than 18% [3,4]. Thus, understanding the molecular biology of NSCLC may help us formulate new therapies.

Hypoxia in the tumor microenvironment has been extensively studied for decades and found to be critical for driving the tumorigenesis of NSCLC [5–7]. Hypoxia inducible factor (HIF) is a master regulator of the hypoxic response and plays a key role in regulating cancer progression and metastasis [8]. Accumulating evidence has shown that HIF has novel effects on epithelial–mesenchymal transition (EMT), which is one of the key steps of metastasis [9]. Therefore, targeting HIF pathway is considered as therapeutic opportunity for cancer treatment.

Dehydroeffusol (DHE) is a phenanthrene compound isolated from the Traditional Chinese Medicine (TCM) Juncus effusus. *In vitro* and *in vivo* biological evaluation reveal that DHE is a bioactive phytochemical with broad activities, including antimicrobial [10,11], anxiolytic and sedative [12] and anti-spasmogenic [13]. In recent years, DHE has been demonstrated to possess anticancer effects through several cancer-associated signaling pathways, such as NF-κB, β-catenin, and endoplasmic reticulum stress [14–17]. DHE effectively inhibits the viability and EMT in neuroblastoma cells [16]. DHE was found to inhibit gastric cancer cell growth and proliferation, as well gastric cancer cell-mediated vasculogenic mimicry and tumorigenicity [14,15]. However, its potential effects on NSCLC remain unknown. Therefore, the objective of the present study was to investigate the effect of DHE on hypoxia-induced EMT in NSCLC cells, as well as the underlying mechanism.

## Materials and methods

### Cell culture and treatments

Human NSCLC cell line (A549 cells) obtained from the (American Type Culture Collection, ATCC, Manassas, VA) were cultured in RPMI-1640 medium (Hyclone, Logan, UT, U.S.A.) with 10% fetal bovine serum (FBS; Invitrogen, Carlsbad, CA, U.S.A.) and 1% penicillin/streptomycin (Sigma-Aldrich, St. Louis, MO, U.S.A.).

Cells in control group were maintained in a normoxic condition. Cells in the hypoxia-induced group were exposed to hypoxia condition (1% O_2_) for 7 days as previously described [18]. Cells in the DHE treatment groups were treated with different concentrations of DHE (10, 20 and 40 μM) for 24 h. Cells in the LiCl treatment group were pretreated with LiCl (10 μM; Sigma) for 2 h, followed by DHE treatment.

### Cell viability assay

A549 cells (5 × 10^3^ cells/well) were seeded into 96-well culture plates and then treated with different concentration of DHE (0, 10, 20, or 40 μM) under a normoxic or hypoxic condition. After indicated incubation time points, 20 μl of MTT (5 mg/ml; Sigma) was added to each well for 4 h. Then the supernatant was discarded, and the formazan crystals were solubilized with 150 μl of dimethyl sulfoxide (DMSO). Subsequently, the absorbance at 490 nm was measured using a microplate reader (Bio-Rad, Hercules, CA, U.S.A.) and expressed as percentages relative to untreated controls.

### Cell migration and invasion assays

Transwell assays were performed using standard protocol with transwell chambers (Corning Inc., Lowell, MA, U.S.A.). A549 cells with 200 μl serum-free medium at the density of 2.5 × 10^4^ cells were seeded in upper chamber. The lower chamber was filled with 600 μl medium with 20% FBS. After incubation for 24 h, the migrated/invaded cells to the lower side of the inserts were fixed with 5% paraformaldehyde and stained with 0.1% Crystal Violet. The cells number from six randomly selected fields was calculated under an inverted microscope (magnification × 200).

### Real-time quantitative PCR analysis

Total RNA was isolated from A549 cells using Trizol reagent (Invitrogen). Reverse transcription was performed to synthesized cDNA using the total RNA and a First Strand cDNA Synthesis Kit (Roche Diagnostics, Mannheim, Germany). Quantitative determination of HIF-1α mRNA level was conducted by real-time RT-PCR with SYBR Green Master Mix (Toyobo, Osaka, Japan). HIF-1α, forward primer: 5′-CAGAGCAGGAAAGAGAGTCATAGAAC-3′, reverse primer: 5′-TTTCGCTTCCTCTGAGCATTC-3′; vimentin, forward primer: 5′- TGAAGTGGATGCCCTTAAAGGAA-3′, reverse primer: 5′- GCAGGCGGCCAATAGTGTCT-3′; snail, forward primer: 5′-CACCTCCAGACCCACTCAGATGT-3′, reverse primer: 5′-GCAGGGACATTCGGGAGAAGGT-3′; slug, forward primer: 5′-GCGAACTGGACACACATACAGTG-3′, reverse primer: 5′-GCTGAGGATCTCTGGTTGTGGT-3′; β-actin, forward primer: 5′-CTCTTCCAGCCTTCCTTCCT-3′, reverse primer: 5′-AGCACTGTGTTGGCGTACAG-3′.

### Western blotting analysis

Total protein was extracted from A549 cells using RIPA lysis buffer (Beyotime, Beijing, China) and then the concentration was measured by BCA Protein Assay Kit (Beyotime). Approximately 50 μg protein samples were processed for Western blotting analysis as described in detail elsewhere [18]. Primary antibodies against E-cadherin, N-cadherin, HIF-1α, β-catenin, p-β-catenin, cyclinD1, c-myc and β-actin were obtained from Abcam (Cambridge, MA, U.S.A.) and used at the recommended dilutions.

### Statistical analysis

Data are presented as the mean ± standard deviation (SD). Statistical analysis was performed using SPSS statistical software 18.0 (SPSS, Inc., Chicago, IL, U.S.A.) with Student’s *t*-test or one-way analysis of variance (ANOVA). *P*<0.05 was considered as statistically significant difference.

## Results

### DHE inhibits the viability of NSCLC cells cultured in normoxic and hypoxic condition

**Figure 1:**
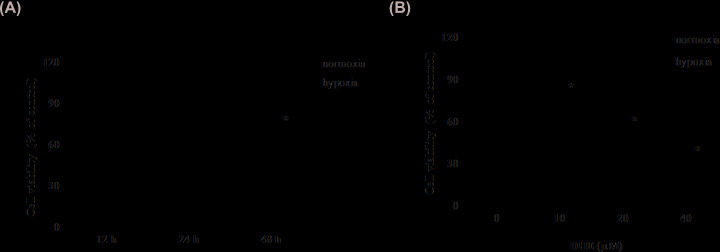
Inhibitory effect of DHE on NSCLC cells viability MTT assay was performed following different treatments. (**A**) Effect of cultured condition on NSCLC cell viability. A549 cells were maintained under normoxic or hypoxic condition for 12, 24, or 48 h; *P<0.05. (**B**) Effect of DHE on cell viability under normoxic or hypoxic condition. A549 cells were treated with different concentrations of DHE (10, 20, 40 μM) for 24 h and exposed to normoxic or hypoxic condition; *P<0.05.

Initially, we evaluated the influence of hypoxia on A549 cells. A549 cells were maintained under normoxic or hypoxic condition for 12, 24, or 48 h. We found that cell viability was markedly decreased after incubation for 48 h under hypoxic condition (Figure 1A). Thus, the cells were exposed to hypoxic condition for 24 h in the subsequent experiments.

Next, we investigated the effect of DHE on A549 cells viability under normoxic or hypoxic condition. A549 cells were treated with different concentrations of DHE (10, 20, 40 μM) for 24 h and exposed to normoxic or hypoxic condition. As shown in Figure 1B, DHE exhibited inhibitory effect on A549 cells viability both in normoxic and hypoxic condition. And the inhibitory effect of DHE is higher in hypoxia-treated A549 cells than that of in normoxia-treated A549 cells.

### DHE prevents hypoxia-induced migration and invasion of NSCLC cells

**Figure 2:**
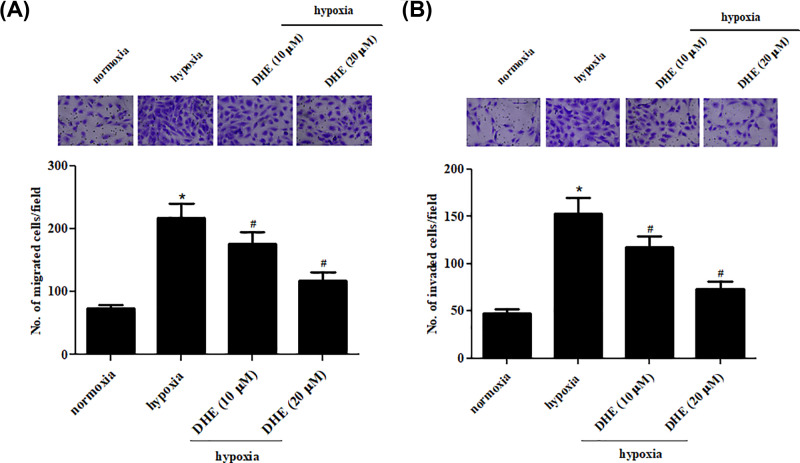
DHE suppressed the migration and invasion of NSCLC cells Untreated A549 cells under hypoxia condition and A549 cells treated with 10 and 20 µM of DHE for 24 h under hypoxic condition. Transwell assays were performed to explore the effects of DHE on A549 cells migration and invasion. (**A**) Quantification of the number of migrated cells. (**B**) Quantification of the number of invaded cells. *P<0.05 vs. A549 cells under normoxic condition. ^#^*P*<0.05 vs. A549 cells under hypoxic condition

Next, we performed transwell assays to explore the effects of DHE on A549 cells migration and invasion. Untreated A549 cells under hypoxia condition and A549 cells treated with 10 and 20 µM of DHE for 24 h under hypoxic condition. The results revealed that migration and invasion abilities were dramatically increased in hypoxia-induced A549 cells as compared with the A549 cells under normoxic condition (Figure 2A,B). Treatment with DHE exhibited dose-dependent inhibitory effect on the migration and invasion abilities.

### DHE inhibits hypoxia-induced EMT in NSCLC cells

**Figure 3:**
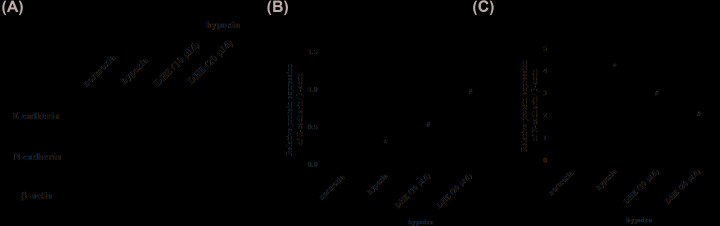
Inhibition of hypoxia-induced EMT by DHE treatment in NSCLC cells A549 cells were treated with 0, 10, and 20 μM of DHE for 24 h under hypoxic condition. (**A**) Western blotting analysis was performed to detect the expression levels of EMT markers including E-cadherin and N-cadherin. (**B** and **C**) Quantification analysis of E-cadherin and N-cadherin. *P<0.05. vs. A549 cells under normoxic condition. ^#^*P*<0.05 vs. A549 cells under hypoxic condition.

Then, we determined the effects of DHE on hypoxia-induced EMT in A549 cells. The cellular morphology of A549 cells was shown in Supplementary Figure S1A. The expression levels of EMT markers including E-cadherin and N-cadherin were detected. Western blotting analysis indicated that exposure to hypoxia caused a significant decrease in E-cadherin expression and an increase in N-cadherin expression. However, these effects were reversed by DHE (Figure 3). In addition, we also found that DHE significantly inhibited the mRNA expression levels of vimentin, snail, and slug in NSCLC cells exposed to hypoxia (Supplementary Figure S1B–D).

### DHE suppresses hypoxia-induced HIF-1α expression in NSCLC cells

**Figure 4:**
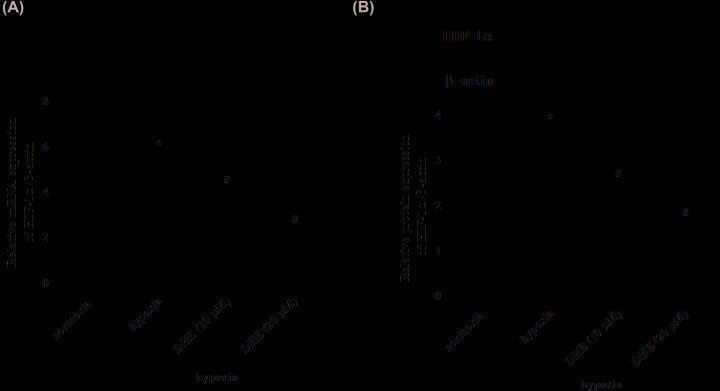
Inhibitory effect of DHE on hypoxia-induced HIF-1α expression in NSCLC cells A549 cells were treated with 0, 10, and 20 μM DHE for 24 h under normoxic or hypoxic condition. The mRNA and protein levels of HIF-1α in A549 cells were respectively determined using qRT-PCR (**A**) and Western blot (**B**). *P<0.05 vs. A549 cells under normoxic condition. ^#^*P*<0.05 vs. A549 cells under hypoxic condition.

Subsequently, we aimed to explore whether HIF signaling was involved in the effects of DHE. The protein and mRNA levels of HIF-1α were respectively determined using Western blot and qRT-PCR. The hypoxia-induced mRNA HIF-1α expression level was significantly suppressed by DHE (Figure 4A). As it was expected, the elevated protein level of HIF-1α was also reduced after treatment with DHE (Figure 4B).

### DHE inhibits the activation of Wnt/β-catenin pathway in NSCLC cells in response to hypoxia

**Figure 5:**
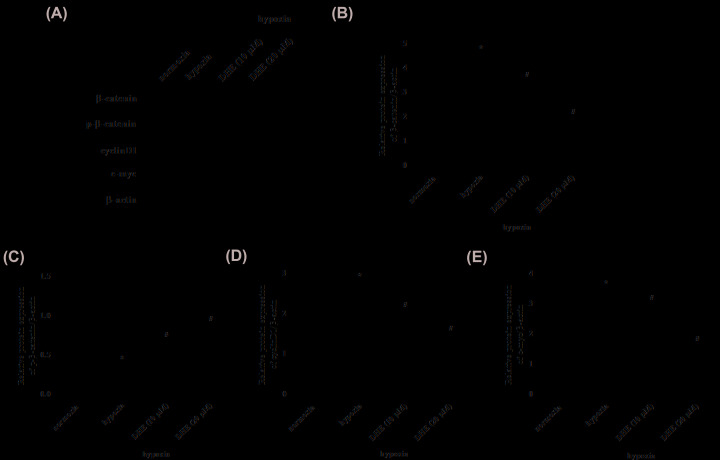
Inhibition of hypoxia-induced activation of Wnt/β-catenin pathway by DHE in NSCLC cells (**A**) To determine the involvement of Wnt/β-catenin pathway in the effects of DHE, Western blotting analysis was performed to detect the expression levels of β-catenin, p-β-catenin, cyclinD1 and c-myc. (**B–E**) Quantification analysis of β-catenin, p-β-catenin, cyclinD1 and c-myc. *P<0.05. vs. A549 cells under normoxic condition. ^#^*P*<0.05. vs. A549 cells under hypoxic condition.

Available literature has demonstrated that Wnt/β-catenin pathway plays a crucial role in HIF signaling [19,20]. Hence, we determined the involvement of Wnt/β-catenin pathway in the effects of DHE. Western blotting analysis elucidated that the expression levels of β-catenin, cyclinD1, and c-myc were obviously induced by hypoxia exposure. However, the increased levels of β-catenin, cyclinD1 and c-myc were mitigated by DHE treatment in a dose-dependent manner (Figure 5). Additionally, DHE could up-regulated p-β-catenin expression in hypoxia-stimulated A549 cells.

### LiCl reverses the effect of DHE on hypoxia-induced EMT in NSCLC cells

**Figure 6:**
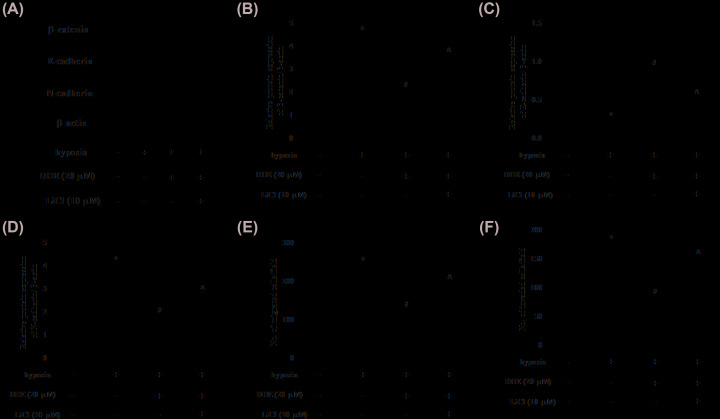
Activation of Wnt/β-catenin pathway reversed the effects of DHE on EMT A549 cells were pretreated with LiCl (10 μM) for 2 h, followed by incubation with 20 μM of DHE for 24 h under hypoxic condition. (**A**) Western blotting analysis was performed to detect the expression levels of β-catenin, E-cadherin, and N-cadherin. (**B–D**) Quantification analysis of β-catenin, E-cadherin, and N-cadherin. (**E** and **F**) Cell migration and invasion were measured. *P<0.05 vs. A549 cells under normoxic condition. ^#^*P*<0.05 vs. A549 cells under hypoxic condition. ^&^*P*<0.05 vs. A549 cells treated with DHE+hypoxia.

Then we utilized a pharmacological activator of Wnt/β-catenin pathway, LiCl, to further verify the role of Wnt/β-catenin pathway. A549 cells were pretreated with LiCl (10 μM) for 2 h, followed by incubation with 20 μM of DHE for 24 h under hypoxic condition. Results in Figure 6A–D showed that LiCl could reverse DHE-caused increased E-cadherin expression and decreased the expression levels of N-cadherin and β-catenin in A549 cells. In addition, LiCl also reversed the effects of DHE on cell migration and invasion in A549 cells under hypoxia condition (Figure 6E,F).

## Discussion

Hypoxia is a common condition presented in tumor environment due to the fast growth of tumor cells and inadequate blood supply [21]. The hypoxia condition contributes to the stemness activity, cancer initiation, and progression. EMT is a novel process that cancer cells lose cell-to-cell contact and gain motility and regarded as the first step in the process of metastasis [22,23]. EMT is characterized by lose of epithelial cells and acquisition of mesenchymal characteristics [24]. Our results showed that DHE significantly inhibited the cell viability of A549 cells in a dose- and time-dependent manner. Moreover, A549 cells were more sensitive to DHE under hypoxic condition compared to the A549 cells under normoxic condition. To determine the effect of DHE on NSCLC metastasis, we used transwell assays to examine cell migration and invasion. The results showed that hypoxia-induced increased migration and invasion abilities were mitigated by DHE in A549 cells. Besides, treatment of DHE caused increased expression level of E-cadherin and decreased expression level of N-cadherin, implying that DHE suppressed the EMT progression. Collectively, DHE inhibited metastasis of NSCLC *in vitro*.

HIF signaling is crucial for the regulation of genes involved in angiogenesis, glycolytic metabolism, and other biological mechanisms in tumorigenesis [25]. There is increasing evidence that hypoxia HIF signaling also plays an important role in regulating cancer metastasis, specifically in multiple key steps of metastasis, including EMT, invasion, extravasation, homing, and pre-metastatic niche formation [6,8,26]. The most studied HIF is HIF-1, which is a heterodimer with two forms, α and β. The form α is expressed in manner oxygen dependent, while the form β is expressed constitutively [21]. Our results proved that DHE suppressed hypoxia-induced increase in both protein and mRNA levels of HIF-1α expression in A549 cells. The data indicated that DHE suppressed HIF signaling under hypoxic condition, which may contribute to the inhibitory effects of DHE on NSCLC metastasis.

HIF is sufficient to induce EMT and invasion through both direct and indirect mechanisms [27–29]. For example, HIF directly induces the transcription of multiple genes, such as Snail, ZEB1, TWIST, and TCF3. Additionally, HIF indirectly promotes EMT via other signaling pathways, such as TGF-β, Notch, and Wnt signaling pathways. The Wnt signal transduction cascade is a main regulator of development in mammals [30]. The canonical Wnt/β-catenin pathway has been found to be involved in diverse diseases and has therapeutic potential [30,31]. Until now, the role of Wnt/β-catenin in tumorigenesis has most prominently been described [32,33]. Zhang et al. [34] reported that HIF-2α promotes tumor progression of pancreatic cancer and has crosstalk with Wnt/β-catenin signaling. Liu et al. [35] demonstrated that HIF-1α and Wnt/β-catenin signaling pathways promote the invasion of hypoxic gastric cancer cells. Additionally, interplay between VHL/HIF-1α and Wnt/β-catenin pathways has been observed in colorectal tumorigenesis [36]. In our research, we found that DHE inhibited the hypoxia-induced activation of Wnt/β-catenin pathway in NSCLC cells. Activation of Wnt/β-catenin pathway by LiCl reversed the inhibitory effect of DHE on hypoxia-induced EMT in NSCLC cells.

In conclusion, our results demonstrated that DHE prevented hypoxia-induced EMT in NSCLC cells by inhibiting the activation of Wnt/β-catenin pathway. These findings suggested that DHE might be a therapeutic target for the metastasis of NSCLC.

## Supplementary Material

Supplementary Figure S1

## Abbreviations

DHE: dehydroeffusol
EMT: epithelial–mesenchymal transition
HIF-1α: hypoxia inducible factor-1α
NSCLC: non-small cell lung cancer
SCLC: small cell lung cancer
TCM: Traditional Chinese Medicine
