# Supplementary material for: Dehydroeffusol inhibits hypoxia-induced epithelial–mesenchymal transition in non-small cell lung cancer cells through the inactivation of Wnt/β-catenin pathway
Source: Biosci Rep. 2020 May 28;40(5):BSR20194284. doi: 10.1042/BSR20194284 (PMC7256677; doi:10.1042/BSR20194284)
Supplement: Supplementary Figure S1 [file BSR-2019-4284_supp.pdf]

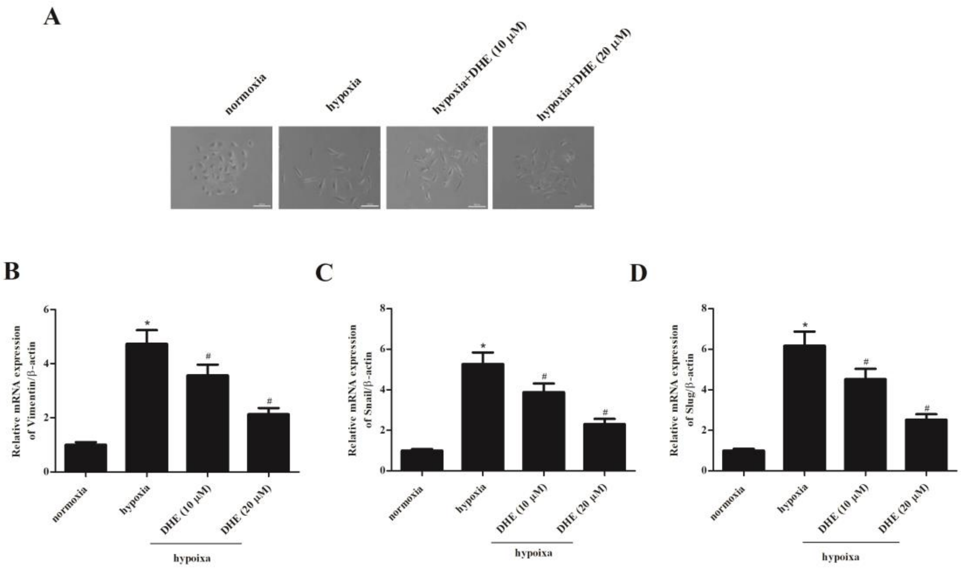

A
normoxia
hypoxia
hypoxia+DHE (10 μM)
hypoxia+DHE (20 μM)
B
Relative mRNA expression of Vimentin/β-actin
normoxia
hypoxia
DHE (10 μM)
DHE (20 μM)
hypoixa
C
Relative mRNA expression of Snail/β-actin
normoxia
hypoxia
DHE (10 μM)
DHE (20 μM)
hypoixa
D
Relative mRNA expression of Slug/β-actin
normoxia
hypoxia
DHE (10 μM)
DHE (20 μM)
hypoixa

Supplementary Figure 1. DHE inhibited the mRNA expression levels of vimentin, snail and slug in NSCLC cells exposed to hypoxia. Untreated A549 cells under hypoxia condition and A549 cells treated with 10 & 20 µM of DHE for 24 h under hypoxic condition. (A) The cellular morphology of A549 cells. (B-D) qRT-PCR analysis was performed to detect the mRNA expression levels of vimentin, snail and slug. *p< 0.05.vs. A549 cells under normoxiccondition.$^{\#}p$< 0.05.vs. A549 cells under hypoxic condition.
